# A system‐based intervention to reduce Black‐White disparities in the treatment of early stage lung cancer: A pragmatic trial at five cancer centers

**DOI:** 10.1002/cam4.2005

**Published:** 2019-02-04

**Authors:** Samuel Cykert, Eugenia Eng, Paul Walker, Matthew A. Manning, Linda B. Robertson, Rohan Arya, Nora S. Jones, Dwight E. Heron

**Affiliations:** ^1^ Division of General Medicine and Clinical Epidemiology The Center for Health Promotion and Disease Prevention The Lineberger Cancer Center The University of North Carolina School of Medicine The University of North Carolina at Chapel Hill Chapel Hill North Carolina; ^2^ Department of Health Behavior The Gilling's School of Global Public Health Chapel Hill North Carolina; ^3^ Leo Jenkins Cancer Center Brody School of Medicine ‐ East Carolina University Greenville North Carolina; ^4^ Cone Health Cancer Center Greensboro North Carolina; ^5^ UPMC Hillman Cancer Center Pittsburgh Pennsylvania; ^6^ Palmetto Health and the University of South Carolina School of Medicine Columbia South Carolina; ^7^ The Partnership Project Greensboro North Carolina; ^8^ Department of Radiation Oncology UPMC Hillman Cancer Center Pittsburgh Pennsylvania

**Keywords:** cancer disparities, health equity, intervention, pragmatic trial, systems change

## Abstract

**Background:**

Advances in early diagnosis and curative treatment have reduced high mortality rates associated with non‐small cell lung cancer. However, racial disparity in survival persists partly because Black patients receive less curative treatment than White patients.

**Methods:**

We performed a 5‐year pragmatic, trial at five cancer centers using a system‐based intervention. Patients diagnosed with early stage lung cancer, aged 18‐85 were eligible. Intervention components included: (1) a real‐time warning system derived from electronic health records, (2) race‐specific feedback to clinical teams on treatment completion rates, and (3) a nurse navigator. Consented patients were compared to retrospective and concurrent controls. The primary outcome was receipt of curative treatment.

**Results:**

There were 2841 early stage lung cancer patients (16% Black) in the retrospective group and 360 (32% Black) in the intervention group. For the retrospective baseline, crude treatment rates were 78% for White patients vs 69% for Black patients (*P* < 0.001); difference by race was confirmed by a model adjusted for age, treatment site, cancer stage, gender, comorbid illness, and income‐odds ratio (OR) 0.66 for Black patients (95% CI 0.51‐0.85, *P* = 0.001). Within the intervention cohort, the crude rate was 96.5% for Black vs 95% for White patients (*P* = 0.56). Odds ratio for the adjusted analysis was 2.1 (95% CI 0.41‐10.4, *P* = 0.39) for Black vs White patients. Between group analyses confirmed treatment parity for the intervention.

**Conclusion:**

A system‐based intervention tested in five cancer centers reduced racial gaps and improved care for all.

## 1 INTRODUCTION

Untreated, early stage, non‐small cell lung cancer is nearly always fatal within 4 years of diagnosis.^1^ Lung resection surgery remains the recommended treatment for cure although stereotactic radiation has been deemed acceptable for a select group of patients.^2^ Despite the high mortality and progressive physical limitations that decisions against treatment portend,^1^, ^3^ Black patients undergo surgery less often than similar White patients. Studies spanning decades^1^, ^4^, ^5^, ^6^, ^7^ show persistence of surgical disparities with similar gaps emerging for radiation.^8^

The reasons for cancer treatment disparities go beyond socioeconomic status, age, and health status.^1^, ^4^, ^5^, ^9^, ^10^ Lathan et al specifically showed that clinician decision making was a greater contributor to lower surgical rates for Black patients than refusal of surgery.^4^ Alternatively, absolute surgical contraindications were the predominant factors that reduced surgeries for White patients.^4^ In a recent prospective study, investigators linked lower surgical rates for all patients regardless of race to negative perceptions of communication and fears that surgery would lead to poor functional status. When considering only Black patients, this study found that lack of a regular source of care was associated with lower surgical rates suggesting that Black patients, possibly experiencing denial or mistrust, were more likely lost to follow‐up. Notably, Black patients with two or more comorbidities (eg, renal insufficiency and insulin‐dependent diabetes) rarely received surgery while similar White patients often did. The comorbidity issue suggests an implicit bias; clinicians were less willing to tolerate higher risk in treatment decisions affecting racially discordant patients.^5^ Despite the preponderance of observational evidence, there is nearly a complete absence of prospective trials to close these gaps.

For the purpose of considering plausible interventions, these disparities were discussed with the Greensboro Health Disparities Collaborative (GHDC), an academic‐community partnership experienced in community‐based participatory research (CBPR).^11^, ^12^ GHDC is a diverse group by race, ethnicity, profession, religion, and neighborhood of residence. Using CBPR principles, GHDC recommended using a multifaceted intervention encompassing elements of real‐time transparency, race‐specific accountability, and enhanced, patient‐centered communication. In this report, we describe a prospective, pragmatic, trial built on these principles.

## 2 METHODS

This study includes subjects enrolled in two multi‐institutional prospective trials using identical interventions. A total of 238 patients were enrolled in the American Cancer Society (ACS)‐sponsored study, Lung Cancer Surgery: Decisions against Life Saving Care—The Intervention (RSG‐05‐217‐05‐CPPB: PI Cykert). The study sites for the ACS work were cancer centers affiliated with the University of North Carolina, East Carolina University, and the University of South Carolina. The Accountability for Cancer Care through Undoing Racism and Equity (ACCURE) study was sponsored by the National Cancer Institute (Grant # 1R01CA150980‐01A1: PI, Eng & Cykert). The ACCURE study applied the intervention to breast and lung cancer patients. The 122 lung cancer patients enrolled in ACCURE were included. Participating sites for ACCURE were the University of Pittsburgh Medical Center's Hillman Cancer Center (UPMC) and Cone Health Cancer Center in Greensboro, North Carolina (Cone). Given the comparability of the system‐based intervention designs, we provide the following descriptions as if applicable to a single trial. Note that both trials were registered with ClinicalTrials.gov (ACCURE, NCT01954641; ACS, NCT01687738).

### 2.1 Study design and intervention

We performed a 5‐year study to examine the effect of an intervention to reduce disparities in treatment received by Black patients with stages I and II lung cancer. Our study was a pragmatic trial according to the PRECIS‐2 definition^13^; specifically, the patients were community based with broad enrollment criteria, treated by usual care providers in a typical cancer care setting using tools and personnel that fit into routine clinic workflows. The study was approved by the governing institutional review board of each enrollment site.

All consented patients received the intervention which consisted of (1) a real‐time warning system derived from automated uploads from electronic health records (EHRs), (2) feedback to clinical teams on completion of cancer treatments according to race, and (3) a nurse navigator who accessed the warning system on a daily basis.

Because of the ubiquitous nature of EHRs and the pervasiveness of quality improvement (QI) techniques in practice, we felt it was unethical to randomize patients to a control group devoid of data feedback and electronic tools. We instead applied audit and feedback to consented patients and established two statistical, whole population control groups without consented patients. The first included all patients diagnosed with stages I and II non‐small cell lung cancer from the five participating institutions from January 1, 2007 to December 31, 2012 and was used to establish baseline treatment rates and racial differences. The second group was comprised of the concurrent population of stage I and II lung cancer patients at two institutions, UPMC and Cone, diagnosed in 2014 and 2015 who were seen in the intervention period but not consented for the study. UPMC and Cone were chosen as the concurrent sample because their informatics teams were willing to establish automated data feeds for non‐study participants as long as the data sets were devoid of personal identifiers. The concurrent group ensured that improvement, if seen in the intervention, would not represent improvement attributable to secular trends. Variables collected for all groups included gender, age, race, health insurance status, zip code, cancer stage at diagnosis, and comorbid illnesses. As care progressed, we recorded whether patients received surgery or stereotactic radiation and dates of all treatments.

Regarding the real‐time registry, we received automated nightly uploads of EHR data for consented patients’ including appointments for clinician visits, tests, treatments, and procedures. One institution was unable to program automated uploads so a research assistant (RA) updated the registry by hand daily after reviewing the EHR. The registry was programmed to deliver alerts when a patient either missed a scheduled appointment or did not reach an expected milestone in care. The milestones were no follow‐up scheduled within 30 days of the initial visit, no surgery or radiation scheduled within 90 days, and no surgery performed or radiation received within 120 days. The real‐time system was a secure, web‐based umbrella system accessed by the navigator on at least a daily basis. Warnings were delivered to the navigators face page and notations for resolution had to be made within the system. Missed appointments were handled directly by the navigator who would re‐engage the patient then identify and resolve barriers to care whether these were logistical, such as a lack of transportation or difficult finances, or perception‐based, such as mistrust, barrier beliefs (eg, prayer alone can cure disease or surgery makes the disease spread), or miscommunication. Since milestone warnings related more to treatment decisions or clinical inertia, the navigator referred these to the physician champion who engaged the clinical team and discussed possible remedies. Although access to the registry system was limited by site, the appearance of warnings, the workflow, and the recording of warning resolutions was uniform. To monitor intervention fidelity, all warnings and navigator responses were logged within the registry system.

For each cancer center, a practicing oncologist served as a physician champion. The champion made other clinicians and staff aware of the study during usual meetings and, in addition to the milestone warnings above, was responsible for delivering quarterly treatment reports for the cancer center population stratified by race.

### 2.2 Patient enrollment

**Figure 1:**
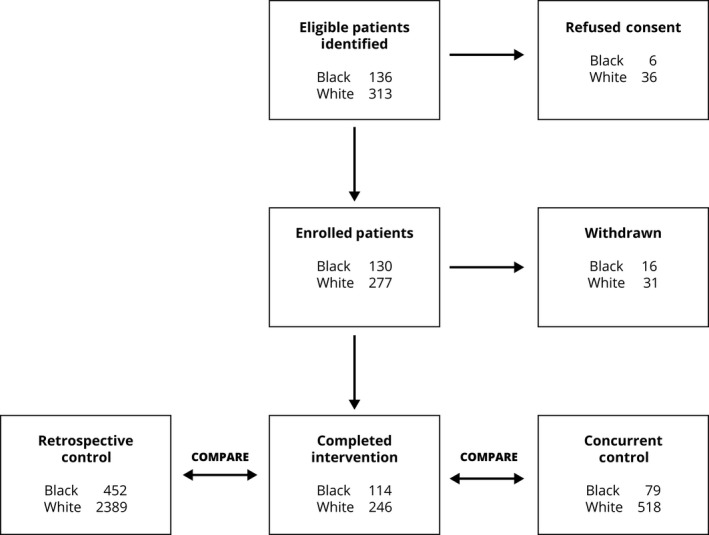
CONSORT Diagram—patient enrollment and progression: a system‐based intervention to reduce Black‐White disparities in the treatment of early stage lung cancer: a pragmatic trial at five cancer centers

Black and White patients with newly diagnosed stage I and II lung cancer between the ages of 18 and 85 were eligible. Exclusions included pregnancy, inability to speak English, and cognitive impairment. Enrollment spanned April 2013 until December 2016. Our goal was to recruit consecutive patients at the participating centers as quickly as possible so that everyone experienced at least 1 year of follow‐up. To identify eligible patients, RA‐screened patient schedules from thoracic surgery, oncology, pulmonary, and multidisciplinary cancer clinics. These schedules were available through the EHR. Most patients screened were ineligible because of a non‐cancer diagnosis, follow‐up for an established diagnosis, a cancer diagnosis other than lung, or stage >2. Figure 1 shows a schematic representation of enrollment and progression through the study. The top row shows the number of eligible patients identified by race and those refusing consent. When eligible patients shared simultaneous appointment times, RA's were trained to prioritize Black patients as a method of oversampling. Informed consent was given by all participants at all sites.

### 2.3 Primary outcome

The primary outcome was receipt of potentially curative treatment (surgical resection or stereotactic radiation); both treatments are superior to no treatment and some reports suggest comparable overall survival.^14^, ^15^ However, since some propensity‐matched comparisons of stereotactic radiation to surgery suggest better outcomes for surgery,^16^, ^17^, ^18^ we evaluated the intervention effect on surgery alone as a secondary outcome. We also examined the use of radiation alone as high rates of radiation exclusive of surgery could suggest inferior care.

### 2.4 Statistical analysis

Patient characteristics including gender, age, median household income by zip code, race, clinical stage, and Charlson score were summarized using descriptive statistics and compared across study groups and within study groups between races using chi‐square and *F*‐tests for categorical and continuous variables, respectively. Separate analyses were performed for the primary outcome (receipt of stereotactic radiation or surgery) and the secondary outcome (receipt of surgery). Since we were interested in estimating treatment differences between Black and White patients within each study group, a logistic regression model was performed for each outcome examining Black compared to White outcomes within each group while controlling for age, income, gender, comorbidities, clinical stage, and site. Because most lung cancer patients were over 65 years old, nearly all study patients had health insurance, so the insurance variable was not entered into models. The retrospective data were used to define baseline treatment rates and the baseline disparity between Black and White patients. Concurrent data were used to follow the disparity during the intervention period to assess secular trends for non‐enrolled patients. For the between group analysis, to control for differences across the three study groups, all data from each group of interest were placed into a single model and study group by race combinations were employed to estimate differences by race and group. Similar logistic regression models were otherwise constructed to those used for within group analyses. As such, we combined and compared estimates of racial differences between baseline (retrospective) and intervention and between concurrent and intervention groups.

## 3 RESULTS

Combining the five study centers, there were 2841 stage I and II lung cancer patients in the baseline, retrospective sample and 16% were Black. Of the 407 patients, 360 originally consented completed the intervention (32% Black). Forty‐seven patients were withdrawn (Figure 1); 14 had advanced disease after further staging; 11 died prior to treatment; 9 had benign biopsy findings; 9 withdrew consent; and 4 were lost to follow‐up. The concurrent control had 597 patients and 13% were Black. Table 1 compares study characteristics among the three groups. Given that the main focus of the study was to examine whether the intervention mitigated Black‐White treatment disparities, demographic comparisons between Black and White patients who were in the intervention group are shown in Table 2. Note that patients are well matched in all demographic categories except Black patients, on average, were younger and had a lower income than their White counterparts.

**Table 1:** Baseline characteristics of the group receiving a multifaceted intervention to improve completion of lung cancer treatment compared to retrospective and concurrent controls

Characteristic	Retrospective controls N = 2841	Intervention group N = 360	Concurrent controls N = 597
Mean age in years (± SD)	68.8 (9.7)^a^	66.2 (9.9)	69.5 (9.2)^a^
Female gender (%)	50.4^a^	44.4	46.7
Black race (%)	15.9^a^	31.7	13.2^a^
Mean household income by zip code ($ ± SD)	66 992 (99 550)	47 065 (15 370)	50 174 (15 392)^a^
Clinical stage II at diagnosis (%)	21.1^a^	16.4	20.4
Mean Charlson score (± SD)	4.2 (3.4)^a^	2.0 (2.6)	3.1 (3.4)^a^

Statistically significant difference (*P* < 0.05) in bivariate comparisons with the intervention group.

**Table 2:** Black‐White comparisons within the intervention group

Characteristic	Black	White	*P*‐value
Gender (percent female)	48.3	42.7	0.32
Clinical stage (percent stage II)	16.8	16.2	0.89
COPD (percent diagnosed)	44.9	45.1	0.96
Charlson score (mean ± SD)	2.0	2.0	0.81
Age^a^mean (± SD)	64.3 (10.1)	67.1 (9.7)	0.01
Median household income by zipcode^a^ mean $ (± SD)	42 300 (13 700)	49 300 (15 600)	0.001

a Difference is statistically significant.

Descriptive analyses were performed for all treatment outcomes. Note the overall treatment rate for surgery or radiation for cure, regardless of race, was 76% in the retrospective group, 96% in the intervention group, and 83% in the concurrent group (all comparisons *P* < 0.001). Differences in surgical treatment also favored the intervention group with a rate of 62% in the retrospective group compared to 76% in the intervention, and 50% in the concurrent group (*P* < 0.001). For Black patients alone, the overall treatment rate was 69% in the retrospective group, 96% in the intervention group, and 79% in the concurrent group. These percentages for White patients followed the same pattern of improvement—78%, 96%, and 84%, respectively. Notably, the radiation only rates in the intervention group were almost identical, 19.1% for Black patients and 21.9% for White patients (*P* = 0.53), suggesting that indications and contraindications for surgery were equally applied to both racial groups in the intervention.

### 3.1 Logistic regression

Note that all regression models controlled for gender, age, median household income by zip code, race, clinical stage, study site, and Charlson score. As expected, age, Charlson score, clinical stage, study site, and income were consistently associated with treatment differences in all the models that included the retrospective and concurrent population groups. However, in the intervention only group, the within group analyses showed that all these factors became moot except for advanced age was associated with less surgery and two study sites strongly favored surgery compared to the other three sites.

### 3.2 Within group comparisons

For the primary outcome, receipt of either of the curative treatments, the crude rate for the retrospective baseline group favored White patients, 78% vs 69% for Black patients (*P* < 0.001); this difference was confirmed in the adjusted model which yielded an odds ratio (OR) of 0.66 for Black patients (95% CI 0.51‐0.85, *P* = 0.001) when compared to similar White patients. When the crude rate for surgery alone was examined, White patients in this baseline group were favored 62% to 59% without statistical significance (*P* = 0.25) in the bivariate comparison. However, in the adjusted model, surgery was significantly lower for Black patients (OR 0.71, 95% CI 0.56‐0.91, *P* = 0.006). The within group comparisons for the intervention cohort did not reveal significant disparities. Receipt of either curative treatment showed crude rates of 96.5% for Black and 95% for White patients (*P* = 0.56). The adjusted analysis, yielded an OR of 2.1 (95% CI 0.41‐10.4, *P* = 0.39) for Black intervention patients compared to similar White patients confirming treatment parity. The comparison within the intervention for the secondary outcome of surgery resulted in a crude rate of 75% for Black and 76% for White patients (*P* = 0.77). The adjusted difference for Black patients receiving surgery alone (OR 0.77, 95% CI 0.41‐1.44, *P* = 0.41) or radiation alone (OR 1.4, 95% CI 0.75‐2.7, *P* = 0.28) was not statistically significant.

### 3.3 Between group comparisons

The between group regression results incorporating the retrospective and intervention groups are shown in Table 3. The race‐group comparisons were all referent to the White retrospective group which served as the gold standard for treatment rates at baseline. Black patients in the intervention actually received curative treatment at a statistically better rate than the White, baseline group. The rate of surgery for Black patients was at least comparable to the White baseline rate. Also of note, overall curative treatment and surgical care were both received at a significantly higher rate in the White intervention group compared to the White baseline suggesting that White patients also benefited from the intervention. The other between group analysis comparing race‐group combinations in the intervention group to White concurrent patients as the referent group is shown in Table 4. The treatment outcomes are consistently statistically superior in the Black and White intervention cohorts compared to concurrent controls, making intervention effect much more likely than a secular trend.

**Table 3:** Between group comparisons among patients with stage I or II non‐small cell lung cancer using race‐group combinations for a model comprised of both retrospective controls and the study intervention group—the White retrospective control is the referent group

Comparison group to White retrospective referent group	Treatment outcome	Percentage treated in intervention group (vs White referent group)	Adjusted odds ratio (95% CI)	*P*‐value
Black intervention	Surgical treatment for cure only	75 (62)	1.2 (0.73, 1.3)	0.5
White intervention	Surgical treatment for cure only	76 (62)	1.6 (1.1, 2.9)	0.008
Black intervention	Surgery or stereotactic radiation for cure	96 (78)	11.9 (2.9, 49)	0.001
White intervention	Surgery or stereotactic radiation for cure	95 (78)	5.8 (3.0, 11)	<0.001
Black intervention	Stereotactic radiation for cure only	22 (16)	2.7 (1.6, 4.8)	<0.001
White intervention	Stereotactic radiation for cure only	19 (16)	1.9 (1.2, 2.9)	0.005

**Table 4:** Between group comparisons among patients with stage I or II non‐small cell lung cancer using race‐group combinations for a model comprised of both concurrent controls and the study intervention group—the White concurrent control is the referent group

Comparison group to White concurrent referent group	Treatment outcome	Percentage treated in intervention group (vs White referent group)	Adjusted odds ratio (95% CI)	*P*‐value
Black intervention	Surgical treatment for cure only	75 (51)	2.6 (1.6, 4.2)	<0.001
White intervention	Surgical treatment for cure only	76 (51)	3.0 (2.1, 4.4)	<0.001
Black intervention	Surgery or stereotactic radiation for cure	96 (84)	5.9 (2.0, 17)	0.001
White intervention	Surgery or stereotactic radiation for cure	95 (84)	4.0 (2.0, 7.9)	<0.001
Black intervention	Stereotactic radiation for cure only	22 (33)	0.68 (0.40, 1.2)	0.17
White intervention	Stereotactic radiation for cure only	19 (33)	0.50 (0.34, 0.74)	0.001

## 4 DISCUSSION

The Institute of Medicine's (IOM) Report on Unequal Treatment, released in 2003, highlighted racial and ethnic disparities across a spectrum of health care services.^19^ Despite the enhanced awareness and the spread of individual‐based, cultural competence training after this report, cancer treatment disparities persist.^20^, ^21^ When focus is placed on early stage, non‐small cell lung cancer, even when analyses are controlled for important confounders such as age, health insurance, socioeconomic status, and comorbid illness, treatment disadvantages for Black patients remain.^1^, ^4^, ^5^, ^21^, ^22^ The surgical treatment gap has remained since its first recognition decades ago and differences in the use of radiation for cure have come to light as the role for stereotactic radiation in lung cancer cure evolves.^8^

The current study used a pragmatic longitudinal trial design at five cancer centers and demonstrated that a multifaceted intervention reduced the Black‐White treatment gap and improved care for patients of both races. What factors attenuated disparities using this approach after the impetus of the IOM report did not? First, the emphasis on race‐specific measurement and feedback must be considered. Cykert et al previously demonstrated that comorbidities seemed to garner worse interpretations for Black patients compared to similar White patients potentially representing implicit bias.^5^ By presenting data to cancer teams according to race and comorbidity, this intervention component provided the transparency needed to highlight unintended trends in care. Second, the intervention design took advantage of widespread availability of digital data through EHRs. Leveraging technology led to a real‐time warning system that flagged patient's missed appointments so that individuals affected by low health literacy, misunderstanding, denial, distrust, or other unanticipated barriers to care could quickly be reengaged. In addition, the same system was programmed to recognize the timing of expected milestones in care. For patients who did not attain these milestones, despite appointment adherence, warnings informed the clinical team about delays related to clinical inertia or communication barriers that could potentially be addressed in real time. It was this component that likely improved treatment for White patients as in our previous work we demonstrated that a segment of the White lung cancer population with early stage disease eschewed surgery due to mistrust, hyper‐religiosity, disbelief of diagnosis, or poor perceptions of communication.^5^ This real‐time registry system mimicked Bickell et al who built and evaluated a registry designed to address racial and ethnic disparities in adjuvant breast cancer care. Bickell's work was successful but required multiple phone calls to clinicians’ offices and hand entry of data to populate the registry.^23^ In the current study, data recognition and entry were automated and, therefore, will be scalable on a whole cancer center population level. Finally, the community discussions performed in partnership with the GHDC led to community input that validated proposed study interventions while enriching navigator training and protocols.

The main limitations of this trial are the patient characteristic differences found between the intervention and control groups. Part of the explanation for these differences is the fact that we intentionally oversampled Black patients in the intervention. Historically, Black lung cancer patients present at a lower median age, have a lower income, and a larger male to female ratio than White lung cancer populations.^1^, ^5^, ^24^ More than doubling the proportion of Black patients represented in this sample may explain nearly all the differences in these three characteristics. Also, comorbidity scores in the retrospective group was obtained by diagnosis counts from cancer registries giving more crude estimates of disease counts and much less specificity than the scores derived from direct clinical data in the other two groups. Nevertheless, in response this demographic variability, all regression models were specifically adjusted to account for all these differences. Also of note, is that we did not adjust for health insurance specifically. The reason for this approach is that less than 5% of our sample were either uninsured or covered by Medicaid creating a cohort almost universally insured. Although private insurance coverage in lieu of or in addition to Medicare could exert a minimal effect on lung cancer treatment decisions, the effect is trivial compared to the substantial difference attributed to uninsurance or Medicaid coverage.^25^ Finally, when focusing specifically on the within group analysis of the intervention group, there's no statistically significant racial difference in overall treatment (which improved 27% for Black patients and 17% for White patients over long documented retrospective rates), surgery (increased 16% and 14%, respectively), or stereotactic radiation—results much different than the within group comparisons in the retrospective group.

In conclusion, treatment for potentially curable non‐small cell lung cancer has been shown for many years to lag behind for Black patients. A multifaceted intervention tested in five cancer centers using the transparency of race‐specific data feedback, real‐time warnings derived from EHRs, and patient‐centered navigation improved care for both Black and White patients while reducing racial differences. Application of this system‐based, pragmatic approach to other cancer treatment disparities at a health system level could have positive effects on treatment completion, treatment equity, and overall outcomes.

## CONFLICT OF INTEREST

Cykert has no actual or potential conflicts of interest to report; Eng has no actual or potential conflicts of interest to report; Walker has no actual or potential conflicts of interest to report; Manning has no actual or potential conflicts of interest to report; Robertson has no actual or potential conflicts of interest to report; Arya has no actual or potential conflicts of interest to report; Jones has no actual or potential conflicts of interest to report; Heron has no actual or potential conflicts of interest to report.
